# Remarkable Similarity in Timing of Absorptive Fine-Root Production Across 11 Diverse Temperate Tree Species in a Common Garden

**DOI:** 10.3389/fpls.2020.623722

**Published:** 2021-01-28

**Authors:** Jennifer M. Withington, Marc Goebel, Bartosz Bułaj, Jacek Oleksyn, Peter B. Reich, David M. Eissenstat

**Affiliations:** ^1^Intercollege Graduate Degree Program in Ecology, Department of Ecosystem Science and Management, The Pennsylvania State University, University Park, PA, United States; ^2^Department of Biology, State University of New York at Oneonta, Oneonta, NY, United States; ^3^Department of Natural Resources, Cornell University, Ithaca, NY, United States; ^4^Department of Silviculture, Faculty of Forestry and Wood Technology, Poznań University of Life Sciences, Poznań, Poland; ^5^Department of Forest Resources, The University of Minnesota, St. Paul, MN, United States; ^6^Institute of Dendrology, Polish Academy of Sciences, Kórnik, Poland; ^7^Hawkesbury Institute for the Environment, University of Western Sydney, Penrith, NSW, Australia

**Keywords:** absorptive fine roots, deciduous, evergreen, precipitation, root phenology, root production, seasonal belowground dynamics, temperate tree species

## Abstract

Long-term minirhizotron observations of absorptive fine roots provide insights into seasonal patterns of belowground root production and carbon dynamics. Our objective was to compare root dynamics over time across mature individuals of 11 temperate trees species: five evergreen and six deciduous. We analyzed the timing and growth on 1st-and 2nd-order roots in minirhizotron images down to a vertical depth of 35 cm, as well as monthly and total annual length production. Production patterns were related to total annual precipitation of the actual and previous year of root production over 6 years. The main or largest peak of annual fine-root production occurred between June and September for almost all species and years. In most years, when peaks occurred, the timing of peak root production was synchronized across all species. A linear mixed model revealed significant differences in monthly fine-root length production across species in certain years (species x year, *P* < 0.0001), which was strongly influenced by three tree species. Total annual root production was much higher in 2000–2002, when there was above-average rainfall in the previous year, compared with production in 2005–2007, which followed years of lower-than-average rainfall (2003–2006). Compared to the wetter period all species experienced a decline of at least 75% in annual production in the drier years. Total annual root length production was more strongly associated with previous year’s (*P* < 0.001) compared with the actual year’s precipitation (*P* = 0.003). Remarkably similar timing of monthly absorptive fine-root growth can occur across multiple species of diverse phylogeny and leaf habit in a given year, suggesting a strong influence of extrinsic factors on absorptive fine-root growth. The influence of previous year precipitation on annual absorptive fine-root growth underscores the importance of legacy effects in biological responses and suggests that a growth response of temperate trees to extreme precipitation or drought events can be exacerbated across years.

## Introduction

Seasonal patterns of fine-root growth often reflect a plant’s ability to capture water and nutrients, to balance competing demands for carbon from different organ systems and to adjust to changing climatic conditions. Compared to aboveground production, seasonal patterns of fine-root production are much less understood, especially with regards to inter- and intra-annual variations of environmental conditions. Thus, improved understanding of the controls over seasonal fine-root growth is important for estimating plant resource partitioning as well as belowground net primary production.

Absorptive roots of trees are non-woody, short-lived, most distal roots to the proximal root attached to the plant stem, exuding primary and secondary metabolites, with very small diameters (e.g., < 1 mm) (McCormack et al., 2015a). They are ephemeral structures that are important for resource acquisition and microbial interactions. Growth of absorptive roots may be influenced by both intrinsic (i.e., endogenous) and extrinsic (i.e., exogenous) factors (Tierney et al., 2003; Comas et al., 2005). For example, intrinsic factors, such as internal competition for carbohydrates amongst plant organs (Farrar and Jones, 2000), can decrease fine-root production during times of production of leaves (Dougherty et al., 1979; Konopka et al., 2005), wood (Côté et al., 2003), or reproductive organs (Comas et al., 2005). Examples of extrinsic factors that can decrease fine-root growth include: low soil moisture (Newman, 1966; Comas et al., 2005), high soil moisture (Leuschner et al., 2001); low soil temperature (Tryon and Chapin, 1983), low solar radiation, (Edwards et al., 2004), and low nutrient availability (King et al., 2002). Interactions of moisture, temperature, and other environmental variables as well as fluctuations of these factors can result in seasonal patterns of high periods (peaks) and low periods (troughs) of fine-root growth.

Surprisingly, the number and scope of studies of fine-root growth periodicity amongst different species is quite limited. Because of challenges to assessing fine-root growth patterns including the large amount of work involved and the time investment to collect, as well as, analyze data, most journal articles on fine-root phenology report only 1 year of data (Lyr and Hoffman, 1967; Hendrick and Pregitzer, 1992; Ruess et al., 1998). In an early study, Sen (1962) reviewed 20 publications on tree root periodicity, with some papers reporting continuous summer growth (one peak) and others a bimodal pattern of annual growth (two peaks). Sen (1962) also reported that root growth periodicity was linked to both intrinsic factors (e.g., the age of the trees, and the species) and extrinsic factors (e.g., environmental conditions like temperature and precipitation) based on the studies he reviewed. However, except for the 3 years of data from McCormack et al. (2014); McCormack et al. (2015b), we are unaware of any studies addressing patterns in annual production across multiple growing seasons and for multiple, mature, even-age temperate tree species with differing leaf phenology and leaf lifespans. Both total annual fine-root production and fine-root growth periodicity are important metrics to understanding the ecology of fine roots.

Over the past 100 years, a variety of root phenology papers have reported on the number of major peaks of root growth observed. There is currently a discrepancy as to the number of peaks and their timing, with many authors reporting one (Brundrett and Kendrick, 1988; Ruess et al., 2003; McCormack et al., 2014), but others reporting two major peaks of root growth (Engler, 1903; Leibundgut et al., 1963; Norby et al., 2004; Noguchi et al., 2005). Various explanations have been proposed to account for a bimodal pattern of root production. For example, during bud break, fine-root growth is often depressed because the shoots may outcompete the roots for carbohydrates (Webb, 1976; Bloom et al., 1985; Joslin et al., 2001), which must all be from stored starch (Keel et al., 2006; Helmisaari et al., 2015; Solly et al., 2018). Later in spring, root growth is often observed to be extremely low during leaf production when again there is a large demand aboveground for carbohydrates (reviewed in Anderson, 2003). Fine-root growth may also be limited by low soil moisture availability in late spring or summer (Newman, 1966; Joslin et al., 2001; Comas et al., 2005; Bauerle et al., 2008), leading to a second root production peak in late summer or autumn generally associated with increased rain and soil moisture (Merritt, 1968) and lower competition for carbohydrates by aboveground organs. Even with seemingly sufficient summer precipitation, high air temperatures and low soil moisture holding capacity can lead to decreased soil water availability in the summer (Lowry, 1962; Weber and Nkemdirim, 1998), thereby, decreasing root production in the middle of the growing season (McDougall, 1916). Reasons for a single peak of fine-root growth during summer include the high demand for water and nutrients and the availability of soil water coupled with warm temperatures. During fall, fewer fine roots are produced because long fine-root lifespans (Withington et al., 2006) result in minimal turnover of roots produced earlier that year, and due to potential competition by mycorrhizal fungi for shared carbohydrates. However, there appears to be no consistent pattern for type of tree (e.g., evergreen vs. deciduous; arbuscular mycorrhizal vs. ectomycorrhizal), location (e.g., N. America vs. Europe), or method of root data collection (e.g., sequential cores vs. minirhizotrons) influencing the root growth patterns observed and reported.

Plant communities typically consist of multiple, interspersed species where identification of fine roots by species has traditionally been difficult, if not impossible. In particular, species identification of roots from minirhizotron images can be problematic because roots are typically not as distinctive as leaves are for identification. For many community-focused or forest ecosystem-focused publications, this is not a problem as the authors research objectives are on stand-level trends. However, it is a challenge for understanding differences in fine-root growth amongst species as well as factors that influence seasonal growth. While many publications over the past 100 years, have focused on one species (Krueger and Trappe, 1967; Ruess et al., 1998; Norby et al., 2004) or were done in mixed-species communities (Brundrett and Kendrick, 1988; Hendrick and Pregitzer, 1992; Joslin et al., 2001), single-species plots are a useful way to overcome issues with root identification while examining species growing in close proximity. Monoculture plots improve our ability to identify species-specific (intrinsic) and or environmental (extrinsic) factors. A common garden setting gives the benefit of plots being spatially in close proximity with similar environmental conditions, while permitting data collection on roots of known species that can be linked to the aboveground production. A shortcoming of this well-established approach is that root growth in single-species plots may not represent that of mixed-species communities, because inter- and intraspecific can result in different patterns of fine-root productivity (González de Andrés et al., 2018; Salahuddin et al., 2018; Zwetsloot et al., 2019).

Previous studies on fine-root growth of temperate tree species report that both intrinsic and extrinsic factors influence fine-root phenology. Abramoff and Finzi (2015) emphasized this idea with a research review of 63 articles covering 25 years on rhizosphere processes across plant species and types (grasses, forbs, and trees). Due to the similarity in extrinsic factors for individuals growing in a common garden, we felt that such a system is necessary for distinguishing between intrinsic and extrinsic factors influencing root growth. For example, if species-specific differences in leaf habit (evergreen or deciduous) are linked to differences in timing of root growth, this would suggest that intrinsic factors like carbohydrate demand dominate control of fine-root growth periodicity. Temperate, evergreen tree species have the capacity to photosynthesize and produce new carbohydrates for a longer period of time during a growing season compared to deciduous tree species (Schulze et al., 1977; Richardson et al., 2010), although their total annual production may be similar (Aerts, 1995), In the spring, evergreen root growth can begin before new leaf growth, combining the usage of stored starch (Endrulat et al., 2010; Epron et al., 2012; Wang et al., 2018) with new photosynthates from existing leaves. The same is true for an extended growing season in the fall, when favorable environmental conditions provide continued carbohydrates for possible belowground root production in evergreens after the deciduous species have dropped their leaves (Wang et al., 2018). In contrast, if timing of root growth is largely independent of leaf habit and better predicted by annual variation in abiotic factors like precipitation, this would suggest that extrinsic factors strongly influence fine-root growth periodicity.

Water availability is an important extrinsic factor influencing fine-root growth. Evergreen species are in general less susceptible to short periods of reduced water availability than temperate deciduous species (Eamus, 1999). The evergreen habit has been repeatedly linked to species that are able to tolerate drier conditions (Aerts, 1995). Because of their lower susceptibility to low water availability, it is reasonable to predict that evergreen species will be able to continue root production during years of lower than average rainfall at a greater level than deciduous species growing in the same environment.

To help address these knowledge gaps of the influence of intrinsic vs. extrinsic factors on root growth, we examined a 6 year data set of fine-root production of mature individuals of 11 temperate tree species. We had three main objectives. First, we examined the influence of leaf habit on timing of root growth. We hypothesized that fine-root growth of evergreen species would consistently occur earlier or later in the growing season than deciduous species, consistent with their longer leaf duration. Our second objective was to determine peak fine-root production in each year for each species and whether one or two peaks of growth occurred. We expected species to vary in root production peaks, based on differences in aboveground leaf habit and different tolerances to and strategies to cope with soil moisture deficits. Third, we determined to what extent total annual fine-root production would be influenced by yearly precipitation, as a major extrinsic factor influencing belowground growth. We hypothesized that tree investment in root growth would be more strongly reduced in years with limited precipitation in the deciduous species compared to the evergreen species.

## Materials and Methods

### Field Site

Our field site was a common garden planting in the Siemianice Experimental Research Forest in west-central Poland, near the village of Biadaszki (51°14.87′N, 18°06.35′E, altitude: 150 m). The field site consisted of two adjacent plantings with 14 species total, nine species per planting, and with four species duplicated between plantings (Szymański, 1982). Species were planted in species-specific, 20 m × 20 m plots in each of three blocks, with a total of 27 plots per planting. Trees were planted at 1 m × 1 m spacing in 1970 (with 1 year-old seedlings) and in 1971 (with 2-year-old seedlings) resulting in mature trees of the same age in the adjacent plantings at the time of our study. Each planting had a fairly uniform topography (quite flat) with very few understory plants due to the high tree density (Withington et al., 2003). Soils were generally nutrient-poor loamy sands (average 80% sand and 15% silt) and classified as fine-loamy, mixed, Mesic Kanhaplic Haplustalfs, and sandy, mixed, Mesic Typic Ustipsamments (Mueller et al., 2012; Supplementary Material S1 for soil type descriptions). For this experiment, we observed root phenology for 11 of the 14 temperate tree species at the site: 5 deciduous species *Acer pseudoplatanus* L., *Acer platanoides* L., *Fagus sylvatica* L., *Quercus robur* L., and *Tilia cordata* Mill.; 1 deciduous gymnosperm *Larix decidua* Mill.; and 5 evergreens *Abies alba* Mill., *Picea abies* (L.) Karst., *Pinus nigra* Arnold, *Pinus sylvestris* L., and *Pseudotsuga menziesii* (Mirbel) Franco. Detailed information regarding descriptions of this site were presented in Reich et al. (2005) and Hobbie et al. (2006).

**FIGURE 1:**
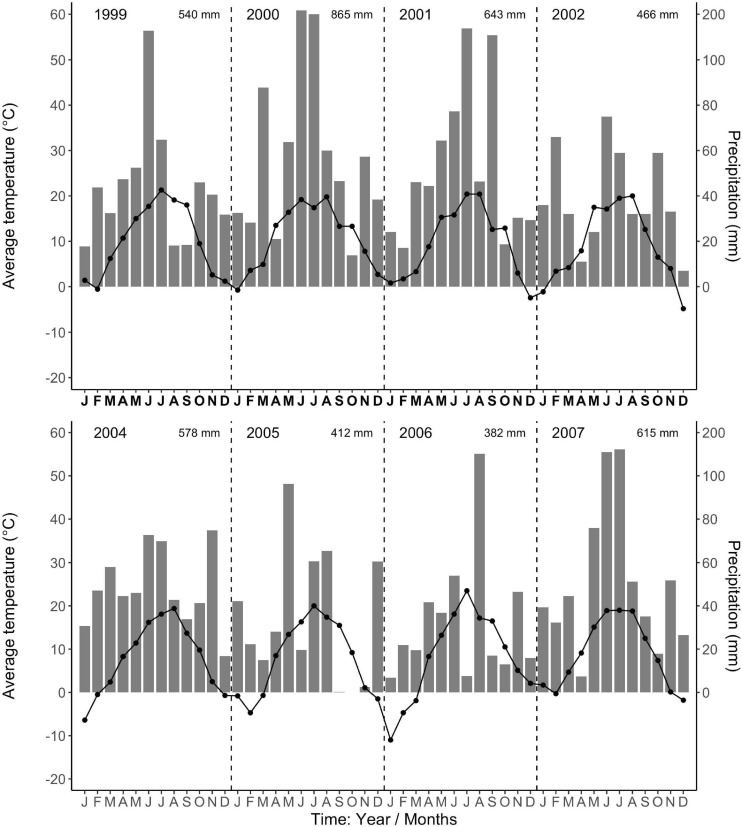
Average monthly air temperatures (°C, point-line) and total monthly precipitation (mm, gray bars) from the Forestry Experimental Station, Siemianice, Poland (1999–2007). The temperature and precipitation data are in fixed proportions, 10°C corresponding to a precipitation of 20 mm, allowing the characterization of periods with indications of water limitation (Gaussen and de Phillips, 1958; Walter and Lieth, 1960). Months where the temperature point exceeds the precipitation bar indicate possible deficit in water availability. The total annual precipitation is stated in the upper right corner for each year. Data for the year 2003 are not available.

**FIGURE 2:**
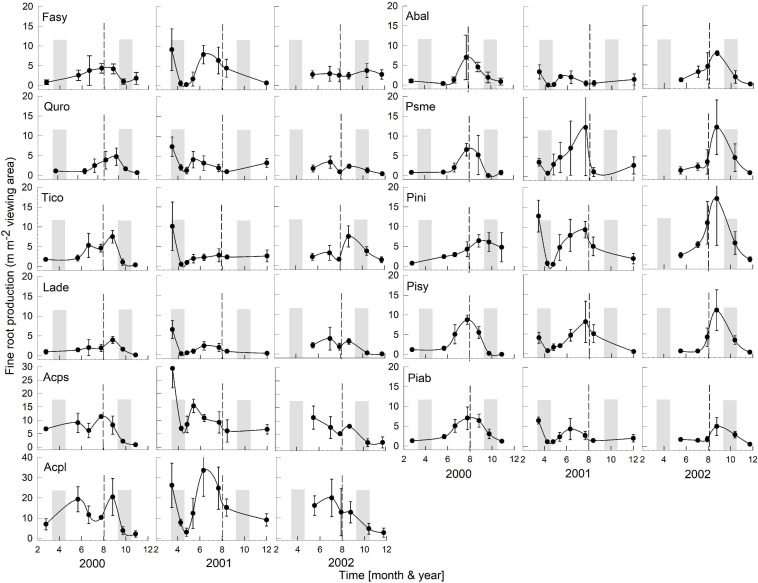
Seasonality of monthly averaged fine-root length production ( ± SE) of 11 temperate tree species growing in a common garden for the years 2000–2002. Species are abbreviated with the first two letters of their genus and species name. Gray bars indicate observed bud break and leaf flush (mid-March to mid-May) as well as leaf fall (mid-September to end of October) of each year. The dashed line is the center of the observed growing season (June–October). Each plot was weighted equally in calculating the seasonal patterns (3–6 plots per species; see section “Materials and Methods” for details). Root production data in this figure were adjusted to 28 day intervals to account for uneven image recording dates within a year. The first data point in each year assumed that root growth did not occur in December or January (see section “Materials and Methods”). Note, the *y*-axis range for all species is 0–20, except for Acps it is 0–30 and Acpl it is 0–40.

**FIGURE 3:**
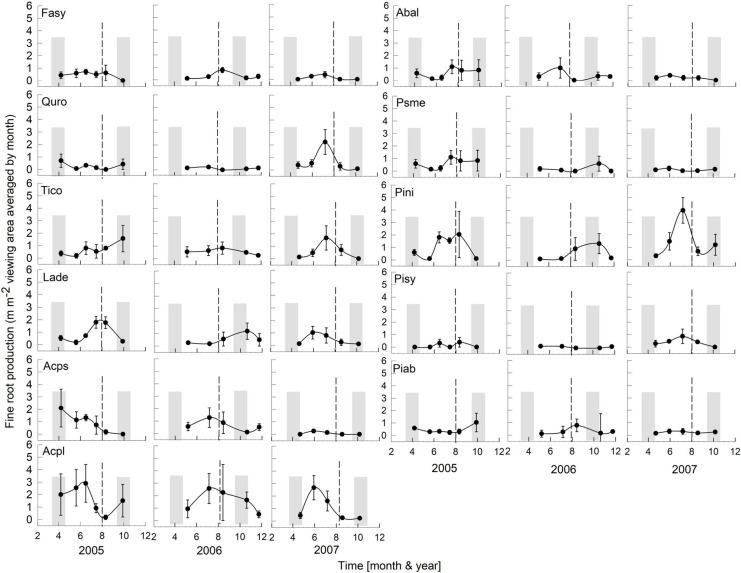
Seasonality of monthly averaged fine-root length production ( ± SE) of 11 temperate tree species growing in a common garden for the years 2005–2007. Species are abbreviated with the first two letters of their genus and species name. Gray bars indicate observed bud break and leaf flush (mid-March to mid-May) as well as leaf fall (mid-September to end of October) of each year. The dashed line is the center of the observed growing season (June–October). Each plot was weighted equally in calculating the seasonal patterns (3–6 plots per species; see section “Materials and Methods” for details). Root production data in this figure were adjusted to 28 day intervals to account for uneven image recording dates within a year. The first data point in each year assumed that root growth did not occur in December or January (see section “Materials and Methods”).

The regional climate is considered transitional between maritime and continental. From 1968 to 1997, the long-term average annual precipitation was 591 mm, with about half falling in 5 months, from May to August (Data from Forestry Experimental Station, Siemianice, Poland). The long-term temperature average was 8.24°C with a mean growing season of about 213 days, calculated as the number of days with an average temperature ≥ 5°C (Szymański and Ceitel, 1989; Ceitel and Wawro, 1999a,b). Over the study period (2000–2007, data for 2003 were unavailable, but see Ciais et al., 2005 about Europe-wide drought in 2003), the average annual precipitation (rainfall, not including snowfall) and temperature were 581 mm and 8.81°C, respectively, very similar to the long-term average. However, the total annual precipitation during the study period varied more than twofold, with a high of 866 mm in 2,000 and lows of 412 mm and 382 mm in 2005 and 2006 (Figure 1), respectively. In 2005 and 2006, the months of low average precipitation coincided with monthly average temperatures of 18°C, resulting most likely in uncommon periods of temporary water limitations in the growing season (Figure 1). Each year in March (1999–2002, 2004), monthly average air temperatures were above 3°C (Figure 1) when bud break occurred (personal observations, J. Withington). Leaf expansion continued with increasing temperatures until about the end of April to mid-May depending on year (personal observations, J. Withington and M. Goebel). Leaf fall in the deciduous species occurred about October each year, when average (24 h/day) air temperatures were about 10°C (Figure 1, gray bars in Figures 2, 3). Needle fall in the evergreens was continuous during the year with peaks in autumn (unpublished data of leaf litter traps, J. Oleksyn).

### Fine Root Production

We examined fine root images collected from 2000 to 2007 which were previously used to establish fine-root lifespan estimates and cumulative root production in a common garden setting (Withington et al., 2003, 2006). Minirhizotron images were collected using a minirhizotron camera and associated image capture software (Bartz Technology Corp., Carpinteria, CA) starting in May 1999, 6 months after tube installation. Images were collected in 1999 at 2–4 week intervals, but sampling intervals were lengthened in 2000–2007 to a 1 month sampling interval due to the extended longevity of the roots of all study species (Withington et al., 2006). Six years of data from 2000 to 2007 are presented in this paper. Data from 1999 are not included: production in the first year was very high for all species, most likely as a result of disturbance from tube installation (Joslin and Wolfe, 1999). Data from 2003 to 2004 are also excluded due to equipment problems leading to minimal and sporadic sampling. In this paper we define seasons based on the days of the Equinoxes and the Solstices.

While the raw data we used (root images) for this current study overlap with two previous papers, our 2003 paper focused on cumulative root production in relation to the minirhizotron tube materials and the 2006 paper focused on median root lifespan and cumulative production. None of these topics are included in this paper. Since the current study examines questions beyond the scope of our original research, certain data that would be useful to the present study (such as more detailed information on shoot phenology and soil temperature measurements) are not available. In addition, while we recognize that mycorrhizae may influence root growth rate (Resendes et al., 2008) and root longevity (McCormack and Guo, 2014), any analysis of mycorrhizal type [e.g., arbuscular (AM) vs. ectomycorrhizae (EM)] on our root production patterns would be compromised because only two species (both *Acer*) were AM.

We focused solely on the first- and second-order absorptive roots, which for our species were < 1 mm diameter. This upper limit is smaller than that used by other researchers, but this limit allowed us to have data which consisted mainly of 1st and 2nd order roots across all 11 of our temperate tree species, but we acknowledge this limit may include some woody roots (Pregitzer et al., 2002; McCormack et al., 2015a). Compared to sequential soil cores, non-destructive technology, such as minirhizotron observation systems and their associated fixed tubes, provide more accurate data on timing of root growth as the measurements are made in the same location over time, removing the issue of confounding temporal variability with spatial variability as well as the confounding effects of multiple soil and root disturbances. Due to the small size of the minirhizotron windows and the majority of roots being first order and, to a limited extent, second order [defining a root in a given order from root tip to its base, not as a segment (*sensu* Withington et al., 2006)], we observed little production of older and higher-order roots in our images.

In a previous study at this field site, we found that tubes made from acrylic plastic provided root standing biomass estimates more consistent with standing crop estimated from soil cores than did tubes made from cellulose acetate butyrate plastic (see Withington et al., 2003); therefore, only observations from acrylic tubes were used in this study. Three tubes were installed per plot, three plots per species in Nov. 1998, keeping the tubes at least 3 m from the plot borders. We used only two *A. alba* plots (six tubes total) because the third plot had become overgrown with a different tree species. *Picea abies* is the one species represented in both plantings with a total of 6 plots. One tube of each of the following species was damaged and lost over the course of the experiment: *A. pseudoplatanus*, *Q. robur*, *T. cordata*, and *L. decidua*, so these species had data from eight tubes total. The minirhizotron tubes were 60 cm long and had an inside diameter of 5.2 cm and a wall thickness of 6.4 mm. The tubes were installed at an angle of 30° from vertical, providing a vertical viewing depth of maximal 35 cm. The tubes were scribed with a strip of 1 × 1.25 cm windows down the upper side. Tops of tubes were wrapped in black electrical tape and sealed with a rubber stopper to keep light and rain out of the tubes.

Root production, numbers of roots per unit viewing area (no. m^–2^), was determined by counting the roots born on each sampling date and summing within plots. If we only used tip counts, this would not be a good comparison of growth across all of our species. Some of the species make very short terminal roots (e.g., *Pinus* spp.) while other make much longer terminal roots (e.g., *Acer* spp.). Therefore, we converted root count to an estimate of root length for each species. The numbers of roots were converted to root length (cm m^–2^) using regression equations of the relationship between number of roots and root length determined for each species (Withington et al., 2006). Briefly, we used a subset of images that included all fine roots present in all tubes for five dates distributed across the years 1999–2003 and representing each season to obtain these regression equations. Root length in each subsample was determined using WinRHIZO Tron (Regent Instruments, Quebec, Canada) and then correlated with the total number of roots in each image (*r*^2^ > 0.88 for all 11 species; Withington et al., 2006).

Dates of root initiation were recorded as the date halfway between the actual sampling date and previous sampling date, with one exception. Beginning in the second year (2000), the time interval from the first sampling date of the year was adjusted to assume all new roots were initiated on 1 February or later. While it has been shown that root growth can occur at air temperatures close to freezing (0°C) (Gaul et al., 2008), we chose to use a more conservative estimate for root growth at air temperatures above 2–3°C (Krueger and Trappe, 1967; Schenker et al., 2014; McCormack et al., 2015b) to be sure we had confidence that root growth was possible in all 11 of our species. Average temperatures of about 3°C occurred in February from 2000–2004 (Figure 1), thus, our choice of 1 Feb. From 2005 to 2007, the average temperatures were lower in February and did not reach this level until March or April, constituting a longer soil freeze; however, we continued to use a February start date to simplify sampling protocols across years.

We calculated absorptive fine-root length production (RLP) (m m^–2^ viewing area) by taking the average across the three tubes per plot, based on total amount of fine roots produced in each plot, allowing for similar weighting of each plot to calculate seasonal patterns (plots are our replicates, not the individual tubes). In 2000, one *F. sylvatica* plot was excluded from the analysis; it contained fewer than five roots total produced over the year and production was too low to accurately assess seasonality when the other plots for the same species had dozens of roots in the same time period (see comment on large error bars in Discussion). These calculations were followed by multiple comparisons, testing differences within a year and differences between years within species.

Absorptive fine-root length production was the response variable of interest and was recorded for each plot at each time point. To analyze the RLP we thus used a linear mixed model, because each plot was repeatedly measured over years and calendar months. Plot was a random effect and species, month, species ^∗^ year, species ^∗^ month were fixed effects in the model. We did not test the interaction (year ^∗^ month) because RLP was only calculated for certain months of actual image collections and therefore it was not appropriate. This analysis was followed by multiple comparison tests, using Bonferroni or Tukey correction when needed.

We also ran linear mixed-effect models to associate total annual RLP to species and annual precipitation (both, current- or previous-year), as well as their interactions (species ^∗^ current- or previous-year precipitation) as fixed effects. Finally, we calculated the correlation coefficient between species annual root production and the total annual precipitation of the previous or current year. For more in-depth resolution, we separated the analysis into monthly periods of the year, in order to identify influential periods of precipitation within a year: (a) the general growing season based on our observed root production 7 months (Apr-Oct), and (b) the last 9 months of the year (Apr-Dec) (Supplementary Table S2). For all statistical analyses we used JMP (version 12, SAS, Cary, North Carolina, United States) and SAS statistical software (Version 9.4; SAS, Cary, North Carolina, United States).

## Results

### Seasonal Pattern of Fine Root Production

Overall, tree species had remarkably similar timing of root growth within a given year. During 6 years of observations, we found the main or largest peak of root production occurred, in general, between June and September (Figures 2, 3), with distinct peaks also occurring sometimes during the spring season (April). In 2000, a main peak occurred in late summer across all 11 species, with evergreen’s peak occurring 1 month earlier (Figure 2, August) than the deciduous species (Figure 2, September). In this year, *A. platanoides* was the only species with two peaks during the growing season. In 2001, an early peak occurred in March for all 11 species, with a second peak occurring in summer (Jun.-Aug.) for six species (*A. platanoides, A. pseudoplatanus, F. sylvatica, P. abies, P. nigra, P. sylvatica*). In 2002, a main peak occurred in September for all five evergreen species and one deciduous species; the two Acer species had early summer peaks, while *F. sylvatica*, *Q. robur* and *L. decidua* did not exhibit a peak in fine-root growth that year (Figure 2). In the last 3 years of observations (2005–2007), a period of extremely low root production, fewer species exhibited a growth peak (five: 2005, eight: 2006, six: 2007, Figure 3). The peaks during the last 3 years were also more variable in magnitude (height of peak) and duration (width of peak), and timing was not as synchronous with peaks spread out from April to October.

We were only able to record spring observations (Feb–Apr.) in 3 years (2000, 2001, 2005) due to winter conditions, such as heavy snow accumulations (Figures 2, 3). We observed a spring peak of root growth, in two of those years, 2001 and 2005. In 2001, which followed an overall very wet year (2000: 865 mm, Figure 1), all 11 species showed an early growth peak in March. In 2005, which followed a year of average precipitation (2004: 578 mm), there was an early peak of root growth only in *A. pseudoplatanus* and *Q. robur*, even though the latter was small, while *A. platanoides* had a broad peak of absorptive fine-root growth from April until the end of June. We noted no evidence that evergreen species had more root growth either earlier in the year or later in the year than deciduous species, even though they are considered capable of photosynthesis during these periods due to their evergreen habit.

After the observational analyses, we quantified the differences in the timing of root length production. Total monthly root length production peaks varied significantly amongst species (*P* < 0.0029), by month (*P* < 0.0001) and by year (*P* < 0.0001) of occurrence (Table 1 and Figures 2, 3). The interaction term species ^∗^ month was not significant, and therefore this term was removed from the model. The effect of year on peak root length production depended on species (interaction species ^∗^ year, *P* < 0.0001). When comparing the interactive effects of species and year, three species contributed to the significant differences in monthly root length production (Table 1) as noted in our qualitative observations above, namely *A. platanoides* and *A. pseudoplatanus*, in the years 2000, 2001, and 2002, and *P. nigra* in addition in the year 2002 (Table 2). These few significant differences are a reflection of the moderate to high variability of fine-root length production (indicated by large standard errors) for individual species. The large standard errors at certain observation dates indicate that timing of peak production in that species varied greatly from plot to plot in that particular year (Figures 2, 3). Conversely, some periods of small standard error, such as typically observed in the months of April and October, indicated very consistent seasonal timing among the plots of that species.

**TABLE 1:** Linear mixed model shows the effect of species, month, and year on root length production [RLP (m m^–2^ viewing area)] from 2000 to 2002 and 2005 to 2007.

***Parameter***	d.f., denDF*	***F*-value**	***P*-value**
Species	10, 25	3.9	<0.0029
Month	10, 1,163	18	<0.0001
Year	5, 1,165	36.9	<0.0001
Species * Year	50, 1,165	3.7	<0.0001

*d.f, degrees of freedom; denDF, denominator degrees of freedom.

**TABLE 2:** Average annual absorptive fine-root length production for each species from 2000 to 2002, and 2005 to 2007.

**Species**	**2000**	**2001**	**2002**	**2005**	**2006**	**2007**	**Total yrs avg.**	2000-2002* **yrs avg.**	**2005-2007**^†^ **yrs avg.**
**(m m^–2^ viewing area)**									

*Acer platanoides*	122 (32)	158 (55)	153 (60)	16 (10)	18 (8.7)	9.8 (0.6)	80 (20)	144 (26)	15 (4.0)
*Acer pseudoplatanus*	77 (14)	112 (18)	83 (33)	9.7 (5.9)	8.6 (5.1)	0.7 (0.5)	48 (12)	91 (13)	6.4 (2.7)
*Fagus sylvatica*	26 (4.0)	38 (6.3)	34 (5.2)	4.2 (0.2)	2.6 (0.1)	1.0 (0.2)	18 (5.1)	33 (7.0)	2.6 (0.5)
*Quercus robur*	21 (7.4)	31 (10)	21 (8.3)	2.9 (1.0)	1.9 (0.8)	5.8 (1.3)	14 (3.5)	25 (4.7)	3.5 (0.8)
*Tilia cordata*	32 (8.2)	32 (12)	33 (8.6)	5.7 (0.9)	6.7 (2.7)	4.6 (1.5)	19 (4.1)	32 (4.9)	5.7 (1.0)
*Larix decidua*	17 (2.1)	17 (4.2)	24 (10)	6.5 (0.5)	5.2 (0.6)	4.1 (1.1)	13 (2.5)	20 (3.7)	5.3 (0.5)
Deciduous Avg.	49 (11)	65 (15)	58 (16)	7.5 (2.0)	7.2 (2.0)	4.3 (0.8)			
*Abies alba*	23 (5.2)	15 (0.2)	30 (5.5)	4.9 (0.7)	5.8 (0.1)	1.9 (0.4)	13 (2.8)	22 (5.7)	4.2 (0.9)
*Picea abies*	36 (5.8)	27 (6.2)	22 (4.4)	4.3 (1.7)	6.5 (2.9)	2.2 (1.1)	17 (2.8)	28 (3.3)	4.2 (1.0)
*Pinus nigra*	36 (6.7)	53 (11)	63 (25)	7.6 (0.6)	4.1 (1.9)	12 (2.7)	29 (6.8)	51 (9.1)	8.0 (1.5)
*Pinus sylvestris*	30 (5.3)	31 (12)	25 (7.0)	0.8 (0.0)	1.4 (0.2)	3.5 (0.4)	15 (4.3)	29 (4.3)	1.3 (0.6)
*Pseudotsuga menziesii*	22 (10)	41 (31)	34 (21)	14 (6.9)	2.2 (1.9)	0.8 (0.8)	19 (6.6)	33 (12)	5.6 (2.9)
Evergreen Avg.	31 (3.4)	33 (6.1)	33 (6.6)	6.5 (1.7)	4.2 (1.0)	4.0 (1.3)			

* 2000–2002. These years had average to above average amounts precipitation.^†^2005–2007. These years had below average precipitation.There were 2, 3, or 6 plots per species with 2 or 3 tubes per plot (see section “*Materials and Methods” for details). Data represent numbers of new roots for each sampling day converted to root length (m m^–2^ viewing area). Standard errors of the mean are given in parentheses.*

### Total Annual Fine Root Production

During the two 3 year periods of observations, the average annual root length production (RLP; m m^–2^ viewing area) was higher in the deciduous species plots than in the evergreen species plots (Table 2); however, this relationship reverses if the values for the *Acer* species are removed. The largest annual average production of total root length per plot for 10 out of 11 species occurred in 2001 and the lowest annual average production was in 2006 or 2007, depending on individual species (Table 2).

There was a notable decrease in total annual RLP between the years 2002 and 2005, with the drought year of 2003 in the interval. The annual production for the first 3 year period was similar within species and much higher than the production for the latter 3 years, which all had lower production. The annual precipitation for 1999–2002 was much higher than for 2003–2007 (Figure 1). Absorptive fine-root growth decreased along with precipitation. The difference in RLP between the wetter years (2000–2002) and the drier years (2005–2007) was striking with a decrease of 73–90% in the deciduous species and 81–96% in the evergreens (Table 2).

When relating RLP with previous year precipitation, our model predicted that in years with reduced precipitation (mean − 1 SD), annual root length production did not differ significantly amongst species. In contrast, in wet years (mean + 1 SD), there were species differences with the strongest influence on RLP by the two maple species (*Acer* spp.) (Table 3).

**TABLE 3:** Summary of parameter estimates predicting individual species’ annual fine-root production (m m^–2^ viewing area) based on the annual average precipitation of the previous years for all 11 temperate tree species for 6 years of observation (2000–2002 and 2005–2007).

	**Fine-root production estimate (m m^–2^)**		
**Species**	**Dry year (−1 SD)**	**Center (mean)**	**Wet year (+ 1 SD)**
*Acer platanoides*	28 a	79.6 a	131.2 a
*Acer pseudoplatanus*	11.6 a	48.4 ab	85.3 b
*Fagus sylvaticus*	4.7 a	17.7 c	30.6 c
*Quercus rober*	4.9 a	14.1 c	23.3 c
*Tilia cordata*	9.7 a	19 c	28.3 c
*Larix decidua*	14.3 a	16.3 c	18.3 c
*Abies alba*	8.4 a	13.3 c	18.2 c
*Picea abies*	10 a	17.1 c	24.1 c
*Pinus nigra*	12.2 a	29.3 bc	46.4 c
*Pinus sylvatica*	9 a	17.6 c	26.1 c
*Pseudotsuga menziesii*	4.8 a	19 c	33.2 c

**Different letters signify significant differences amongst species (P < 0.05). Dry years are indicated by −1 SD and wet years by +1 SD. Production estimates for dry years (−1 SD) showed no significant differences.**

From our linear mixed model, total annual RLP was associated with the actual year’s (*P* = 0.003) and previous year’s precipitation (*P* < 0.001), but only the effect of previous year precipitation on RLP depended on species (interaction species ^∗^ previous year annual precipitation, *P* < 0.014) (Supplementary Table S1). The residual variance component was reduced by 15% when controlling for the precipitation of the current year of root production in the model that included only the main effect of species. However, when controlling for precipitation of the previous year of root production, the model was a much better fit with the residual variance dropping by 59%. Furthermore, the interactions of species and previous year’s precipitation was highly significant and the model including the interaction between previous year precipitation and species reduced the residual variance by 42% compared to the model with species alone (Supplementary Table S1).

To more closely examine the relationship between precipitation and RLP, we categorized species into groups based on leaf habit. Total annual precipitation of the previous year was significantly correlated (*P* < 0.005) with root length production over the 6 years of observations for both groups, the six deciduous and five evergreen species. We found similar significant correlations when grouping the five Angiosperms, as well as smaller groups: the two *Acer* spp., and *Q. robur* + *F. sylvatica* + *T. cordata* together (Table 4). The correlation coefficients range from 28 to 32% for the deciduous and evergreen groups (leaf habit), whereas these coefficients increase to 58–59% in narrower groupings, i.e., the two subgroups of Angiosperms, the *Acer* spp. and *Q. robur* + *F. sylvatica* + *T. cordata*. Total annual precipitation of the actual year was only significantly correlated with RLP for two groups, the five evergreens and *Q. robur* + *F. sylvatica* + *T. cordata*; however, the correlation coefficients were lower than for the previous year precipitation for these groups (Table 4).

**TABLE 4:** Correlation coefficient (*r*^2^) and *p*-values of tree species categories’ annual total fine-root length production (m m^–2^) and annual precipitation data.

**Species’ categories**	**Actual 12 months**	**Previous 12 months**
Angiosperms	n.s.	0.28 *0.002*
Quro, Fasy, Tico	0.23 0.04	0.59 *0.0002*
Acpl, Acps	n.s.	0.58 *0.004*
Deciduous Angio + Lade	n.s.	0.25 *0.002*
Evergreens	0.15 0.019	0.32 *0.0003*

**Italics indicate P < 0.05; n.s, not significant. Species are abbreviated with the first two letters of their genus and species name. Angio: Angiosperms axis range for all species is 0–10, for Acpl it 0–20.**

To determine if there was a particular seasonal time period of the influence of precipitation on annual RLP, we also considered precipitation in time periods less than the full year. For the previous year, both the precipitation of the growing season (7 months, Apr-Oct) and of the last 9 months were significantly correlated with RLP (all categories, *P* < 0.01) over the 6 years of observations. The correlation coefficients for the partial years were similar to the 12 month correlation for all of the groups (Supplementary Table S2). For the actual-year, when looking at the same two time periods, the evergreen group was significant for both, the 7 month and the 9 month precipitation interval (both *P* < 0.05) while the *Q. robur* + *F. sylvatica* + *T. cordata* group was significant for the 7 month period (*P* = 0.03). The correlation coefficients for these three relationships were much lower than the those for the previous year or parts of the previous year but were similar to those for the actual year (Supplementary Table S2).

## Discussion

### Seasonal Patterns of Fine Root Growth

Despite widely different leaf habits and phylogeny, the seasonality of fine RLP was generally synchronized across both evergreen and deciduous tree species within a given year (Figures 2, 3); therefore, our data do not support our hypothesis that evergreens consistently have earlier root growth than deciduous species. After leaf expansion, root production often increased and peaked in June-August or September-October and dropped off later in the year (Figures 2, 3). This alternation of shoot and root growth (or asynchronicity of growth) has been reported repeatedly in studies with seedlings (Reich et al., 1980; Thaler and Pages, 1996; Makoto et al., 2020), with mature trees (e.g., *Q. alba*: Reich et al., 1980), and with mixed biome data sets (Abramoff and Finzi, 2015). However, this pattern of alternation of root and shoot growth in spring is not always the case. Leibundgut et al. (1963) reported almost parallel growth in above- and belowground organs for 1 year-old seedlings of three evergreen and four deciduous species. McCormack et al. (2015b) also reported synchronous growth of leaves and roots in liriodendron tulipifera.

Our 3 years of early spring observations limit general statements about early RLP; however, we observed that root growth can occur before bud break and leaf expansion. We do note that 2000 was the wettest year, which in combination with mild winter temperatures (Figure 1), presumably contributed to the increased peak growth for all our 11 study species in spring 2001. However, the 2005 spring root production (that occurred only in the two *Acer* species) followed an average precipitation year in 2004, along with lower winter temperatures (Figure 1). Together these observations support the view that previous year rainfall can also impact new root growth early in the next year.

While the RLP was often synchronized across species in a particular year, the timing of peak root growth was not consistent from year to year within species, suggesting a strong influence of extrinsic factors. It is difficult to determine which extrinsic factors might have the greatest influence on absorptive RLP, considering all of the species were exposed to similar environmental factors, including precipitation, soil water content and air temperature. In general, our results are consistent with those of McCormack et al. (2012); McCormack et al. (2015b) who reported five of their six study species had large variability in timing of peak root production among years, suggesting that various environmental conditions were influencing the root production peaks.

### One or Two Peaks of Root Production? It Depends

Our data support the commonly held notion of one peak of absorptive fine-root growth during a growing season. Many authors have reported a single root growth peak in the late spring to summer months (Hendrick and Pregitzer, 1992; Ruess et al., 2003; McCormack et al., 2014; Montagnoli et al., 2019), a pattern often linked to warm air and soil temperatures and consistently moist soil conditions. Reich et al. (1980) reported synchrony of root growth flushes across *Quercus* spp. seedlings; however, our study is the first to report that mature tree species individuals with widely different phylogeny and leaf habit have similar root growth patterns in a given year, despite annual variability.

We observed late summer absorptive fine-root growth peaks in 2 years; specifically, in 2000 (four deciduous species) and 2002 (nine deciduous and evergreen species). Moreover, if a late peak occurred, it was the only peak for the year. These data differ from those reported in the literature (e.g., Engler, 1903) and could be due to the limitation of our observations to a depth of 30 cm. Peaks of root growth may occur at different depths during a year; shallow observation depths may not observe second root flushes occurring deeper in the soil. Working with 1–4 year old seedlings in a walk-in rhizotron, Lyr and Hoffman (1967) reported two peaks of growth in eight species, with the second peak in the late summer at depths of about 1 m and deeper. Münzenberger et al. (2003) also recorded a second growth peak in autumn across several species at depths of more than 1 m. Burke and Raynal (1994) found a single fine RLP peak at 0–10 cm, between June and September, and two root production peaks at deeper depths (10–20, 20–30, 30–40, and 40–50 cm).

Tierney et al. (2003) proposed that the number of root growth peaks is related to the geographical location of the study site and the presence or absence of summer drought conditions. They observed in a temperate forest that fine-root production tracked temperatures with usually one peak in mid-summer. Our root production data do not support their conclusion; rather, our data suggest that linking root peak production and summer droughts may not always be true. We observed one peak of production for 5 years in Poland (Figures 2, 3); however, in 2001 (wettest year), six species had two peaks of growth with the second peak in summer (Jun–Aug.). There were no second peaks observed in the drier observation years (2005–2007). Other studies in geographically similar locations like Germany, Switzerland and Denmark (Resa, 1877; Büsgen, 1901; Ladefoged, 1939) reported two peaks, with the second in autumn.

### Total Annual Fine Root Production

Our additional objectives were to quantify total annual absorptive fine-root length production (RLP) among species, the phenology of fine-root growth, and to evaluate the linkage between total annual RLP and precipitation. We observed that the six deciduous tree species had greater annual RLP than the five evergreen species (due to the large production in the *Acer* spp.) and that total annual production for both groups varied more than ninefold between the highest production year (2001) and the lowest production year (2007) (Table 2). We also found total annual RLP was low in 2005–2007 following the extreme drought year of 2003, when European air temperatures in July reached on average 6°C above the long-term means, and annual precipitation deficits were as high as 300 mm, 50% below long-term precipitation averages (Raspe et al., 2004; Ciais et al., 2005). We suggest that the multiple-year drought conditions caused by below-average precipitation (2003, 2005, 2006; Figure 1) might have contributed to the long-term decline in RLP (Table 2; e.g., Olesinski et al., 2011). It is likely that RLP in 2005–2007 was low due to some combination of the limited vertical depth of observations in our minirhizotron tubes (max. 35 cm) and because the investment in root growth could not be sustained by the trees in the common garden due to chronically low precipitation across multiple years. However, drought does not consistently decrease absorptive fine-root production (Büttner and Leuschner, 1994) but can instead stimulate root production (Teskey and Hinckley, 1981; Leuschner et al., 2001), and this may have occurred at depths below our observation depth. Other extrinsic factors may also influence total annual root production, although we do not have data to evaluate these.

It is unlikely that the low root production in 2007 at the end of the 6 years of observation was due to effects of the acrylic tubes (Withington et al., 2003). Other long term minirhizotron studies did not observe a decrease in root growth after 6 years (Norby et al., 2004; Pritchard et al., 2014). In addition, our observations from mature trees are similar to those of Atkinson (1983) who reported differences of 6–20-fold in yearly maximum and minimum RLP of apple (*Malus domestica*) over 10 years. Atkinson ascribed the differences he observed to planting response and to changes in seedling physiology. However, this reasoning seems to be insufficient for explaining our observations in relatively mature temperate trees.

Our final objective was to calculate average total annual absorptive fine RLP for each species and assess the correlation of RLP with cumulative precipitation over various time periods. We found significant correlations between total annual RLP with current-year precipitation as well as with previous-year’s precipitation for our 11 species. However, when we considering the residual variance, as well as correlation coefficients, previous-year precipitation had a greater effect on fine-root growth compared to current year precipitation (Tables 3, 4). These results underscore the influence of water availability (precipitation, an extrinsic factor) on plant growth, both in the current as well as the previous year. In a study of *Abies balsamea* root growth with respect to drought, Olesinski et al. (2011) also reported the importance of the previous-year’s precipitation at influencing fine-root growth of trees under drought in the current year. Hadegorn et al. (2016) linked the buffering of root growth in beech under short-term drought conditions to the accumulation of carbohydrates in the root system, permitting renewed or sustained root growth after a period of drought stress. Similarly, Mazza et al. (2014) and Rita et al. (2014) reported that in years of decreased precipitation, the influence of a previously wet year had a significant impact on maintaining radial growth. In addition, there is some evidence that photosynthates of spring and summer may remain in the aboveground parts of the trees, whereas photosynthates produced in fall tend to go to the roots, both for direct root growth and for starch storage (Endrulat et al., 2010; Epron et al., 2012; Adams and Eissenstat, 2014). Collectively, these findings suggest that a legacy effect of annual precipitation may often carry over into the next year resulting in continued root growth when the year before had sufficient precipitation, but the actual year may not.

### Environmental Conditions Matter

For temperate trees, species, or leaf habit, exert less influence on root growth dynamics than often assumed, with a greater response from environmental influences. Our results concur with numerous other studies: root growth is plastic to environmental conditions. However, we suggest that the “environmental conditions” linked to absorptive fine-root growth are rarely as simple as some researchers may suggest. This is notable when trying to determine the absorptive fine-root growth patterns of a single species in a specific region (i.e., *Picea abies*). Engler (1903); Ladefoged (1939), and Leibundgut et al. (1963) all report two peaks for *P. abies*, an early spring and autumn peak. Our study found *P. abies* to have only one peak in 4 years (late summer: 2000, 2002, and 2006; autumn: 2005), while only 2001 had two peaks (spring and mid-summer). To reconcile this variability across studies, we consider Engler’s (1903) study conditions. Neither Engler’s nor our site experienced low summer precipitation followed by a wet autumn (conditions generally associated with two annual peaks), yet we observed different patterns in root production for the same species at similar latitudes (Switzerland and Poland). It is possible that the differences in root growth peaks were not due to precipitation differences but to differences in site soil characteristics. Soil characteristics and temperature interact with precipitation to influence soil moisture availability (Lowry, 1962; Weber and Nkemdirim, 1998). Engler’s research site (Adlisberg, 670 m), had a soil that was nutrient-rich and loamy, while our site, at 150 m, has a sandy, relatively nutrient-poor soil. Differences in (a) soil water retention at the two sites, (b) temperature regimes in lower vs. higher elevation, and (c) individual age could all contribute to the discrepancies in fine-root phenology between Engler’s study and our own. The year-to-year variability of seasonal patterns of root growth, as illustrated here, suggests that a more comprehensive understanding with more detailed plant and environmental measurements are needed to adequately predict patterns of root growth in temperate trees.

## Conclusion

The relatively consistent growth patterns across 11 tree species for a particular year underscore the benefit of a multi-species, long-term study for looking at absorptive fine-root production. Our demonstration of relatively synchronous fluctuation in the peak periods of root growth across multiple years for 11 species suggests a large influence of environmental conditions on root growth. The linkage of current year total RLP with previous-year rainfall indicates the importance of buffering resulting in lags in biological responses (i.e., legacy effects), and thus of long-term (>3 years) studies for root phenology. Long-term, species-specific studies with comprehensive environmental and physiological measurements will continue to advance our understanding of how tree species allocate carbon belowground and the relative role of environmental conditions in shaping these dynamics.

## Data Availability Statement

Root data will be submitted to the Fine-Root Ecology Database (FRED) jointly housed through the TRY Plant Trait Database and ORNL DAAC. Iversen CM, Powell AS, McCormack ML, Blackwood CB, Freschet GT, Kattge J, Roumet C, Stover DB, Soudzilovskaia NA, Valverde-Barrantes OJ, van Bodegom PM, Violle C. 2018. Fine-Root Ecology Database (FRED): A Global Collection of Root Trait Data with Coincident Site, Vegetation, Edaphic, and Climatic Data, Version 2. Oak Ridge National Laboratory, TES SFA, U.S. Department of Energy, Oak Ridge, Tennessee, U.S.A. Access on-line at: https://doi.org/10.25581/ornlsfa.012/1417481.

## Author Contributions

JW and DE planned and designed the specific research described herein. PR and JO organized the broader project on-site of which this was a component and facilitated use of the research site. JW, BB, and MG collected and analyzed the data. JO contributed data. JW and MG wrote the manuscript, with assistance from all authors.

## Conflict of Interest

The authors declare that the research was conducted in the absence of any commercial or financial relationships that could be construed as a potential conflict of interest.

## Supplementary Material

Supplementary Table 1 Linear mixed model root length production and annual precipitation.

Supplementary Table 2 Correlation coefficients by species groupings for RLP and each actual and previous year precipitation (9, 7 months).

Supplementary Description 1 USDA Soil type descriptions.
