# Supplementary material for: Remarkable Similarity in Timing of Absorptive Fine-Root Production Across 11 Diverse Temperate Tree Species in a Common Garden
Source: Front Plant Sci. 2021 Jan 28;11:623722. doi: 10.3389/fpls.2020.623722 (PMC7875864; doi:10.3389/fpls.2020.623722)
Supplement: Supplementary Table 1 — Linear mixed model root length production and annual precipitation. [file Data_Sheet_1.docx]

Supplementary Material

# Supplementary Information

Description S1

Characterisation of Haplustalfs and Kanhaplic Haplustalfs based on the USDA soil taxonomy

Soil classification: Haplustalfs are Ustalfs with an argillic horizon. They do not have a kandic or natric horizon, do not have a duripan that has its upper boundary within 100 cm of the surface, do not have a petrocalcic horizon within 150 cm of the surface, and do not have much plinthite. Many of these soils are relatively thin, are reddish to yellowish brown, or have a significant clay decrease within 150 cm of the surface. They are not dark red or dusky red throughout the argillic horizon. The argillic horizon does not have both a clayey or clayey-skeletal particle-size class in some subhorizon in its upper part and an abrupt upper boundary. Haplustalfs are commonly in areas of relatively recent erosional surfaces or deposits, most of them late Pleistocene in age. Some of the soils have a monsoon climate. Others have two, more or less, marked dry seasons during the year. Typic Haplustalfs have a CEC of less than 24 cmol(+)/kg clay (by 1N NH4 OAc pH 7) in 50 % or more either of the argillic horizon if less than 100 cm thick or of its upper 100 cm.

United States Department of Agriculture. 1999. Soil Taxonomy: A Basic System of Soil Classification for Making and Interpreting Soil Surveys, 2^nd^ ed. National Resource Conservation Service, Washington, D.C.

# Supplementary Tables

**Table S1**. Linear mixed model shows the effect of species and annual precipitation (both, current- or previous-year) on total annual root length production (RLP [m m^-2^ viewing area]) from 2000-2002 and 2005-2007.

| ***Parameter*** | **d.f. , sum of squares** | ***F*-value** | ***P*-value** |
| --- | --- | --- | --- |
| Species | 10, 232938 | 6.5 | < 0.0029 |
| Actual year annual precipitation [mm] | 1, 36389 | 10.2 | < 0.0001 |
| Species * actual year annual precipitation [mm] | 10, 24673 | 0.69 | < 0.0001 |
| Previous year annual precipitation [mm] | 1, 139437 | 39.2 | < 0.0001 |
| Species * previous year annual precipitation [mm] | 10, 97946 | 2.8 | 0.013 |

**Table S2**. Correlation coefficients (*r^2^*) and *p*-values of individual species’ fine root length production for each, actual and previous years’ 7-month (Apr-Oct) and 9- (Apr-Dec) annual precipitation. The 6 deciduous species are listed 1^st^.

|  | Actual  years’s precipitation | | | | Previous  years’s precipitation | | |
| --- | --- | --- | --- | --- | --- | --- | --- |
| Species &  categories | | 7 months [Apr - Oct] | 9 months [Apr - Dec] | | 7 months [Apr - Oct] | 9 months [Apr - Dec] | |
| **Angiosperms** | | n.s. | n.s. | | 0.28 *0.0029* | | 0.26 *0.0037* |
| Quro, Fasy, Tico | | 0.26 *0.03* | n.s. | | 0.59 *0.0002* | | 0.56 *0.0003* |
| Two Acers | | n.s. | n.s. | | 0.55 *0.006* | | 0.52 *0.008* |
| Deciduous Angio + Lade | | n.s. | n.s. | | 0.24 *0.0026* | | 0.23 *0.003* |
| **Evergreens** | 0.15 *0.02* | | 0.11 0.04 | 0.31 *0.0005* | | | 0.29 *0.0007* |
